# Ciprofloxacin and Tetracycline Resistance Cause Collateral Sensitivity to Aminoglycosides in *Salmonella* Typhimurium

**DOI:** 10.3390/antibiotics12081335

**Published:** 2023-08-18

**Authors:** Mahadi Hasan, Jun Wang, Juhee Ahn

**Affiliations:** 1 Department of Biomedical Science, Kangwon National University, Chuncheon 24341, Gangwon, Republic of Korea; 202216366@kangwon.ac.kr; 2 College of Food Science and Engineering, Qingdao Agricultural University, Qingdao 266109, China; jwang@qau.edu.cn; 3 Institute of Bioscience and Biotechnology, Kangwon National University, Chuncheon 24341, Gangwon, Republic of Korea

**Keywords:** *Salmonella* Typhimurium, collateral sensitivity, cross resistance, efflux pump, proton motive force

## Abstract

The objective of this study was to evaluate collateral sensitivity and cross-resistance of antibiotic-induced resistant *Salmonella* Typhimurium to various antibiotics. *S*. Typhimurium ATCC 19585 (ST^WT^) was exposed to ciprofloxacin, gentamicin, kanamycin, and tetracycline to induce antibiotic resistance, respectively, assigned as ST^CIP^, ST^GEN^, ST^KAN^, and ST^TET^. The susceptibilities of the antibiotic-induced resistant mutants to cefotaxime, chloramphenicol, ciprofloxacin, gentamicin, kanamycin, polymyxin B, streptomycin, tetracycline, and tobramycin were determined in the absence and presence of CCCP and PAβN. ST^CIP^ showed the cross-resistance to tetracycline and collateral sensitivity to gentamicin (1/2 fold) and kanamycin (1/4 fold). ST^TET^ was also cross-resistant to ciprofloxacin (128-fold) and collateral sensitive to gentamicin (1/4-fold) and kanamycin (1/8-fold). The cross-resistance and collateral sensitivity of ST^CIP^ and ST^TET^ were associated with the AcrAB-TolC efflux pump and outer membrane porin proteins (OmpC). This study provides new insight into the collateral sensitivity phenomenon, which can be used for designing effective antibiotic treatment regimens to control antibiotic-resistant bacteria.

## 1. Introduction

*Salmonella* is a major foodborne pathogen that causes widespread contamination and infections [1]. One of the main serovars that cause human and animal salmonellosis is *Salmonella* Typhimurium [2]. It has been reported to cause 115 million human infections and 370 thousand deaths per year on a global scale [3]. Severe *Salmonella* infections require antibiotic intervention; however, antibiotic resistance in *Salmonella* Typhimurium presents yet another challenge to overcome [2]. Overuse, misuse, and extensive application of antibiotics in clinics and livestock sectors are putting constant selection pressure on the bacteria population, thus giving rise to resistance in pathogenic bacteria, including *S*. Typhimurium [4,5]. Worryingly, the emergence of antibiotic-resistant bacteria is much more frequent than the discovery rates of novel antibiotics [6]. In fact, no new class of antibiotics has been discovered in the last two decades despite substantial efforts. Because of the limited effective antibiotic options available, a rational application strategy of existing antibiotics should be considered with a particular emphasis on suppressing or reversing resistance evolution in pathogenic bacteria [7].

The emergence of cross-resistance in pathogenic bacteria has far-reaching consequences. The cross-resistance occurs when the development of resistance to one antibiotic causes an increase in resistance to another antibiotic in the same class or even against different classes of antibiotics [8]. This phenomenon occurs due to acquired resistance mechanisms, such as genetic mutations of the target site, reduced uptake of the antibiotics, and increased efflux pumps, which confer resistance to one antibiotic and can also confer resistance to other antibiotics with similar structure, function, or target site [9]. The selection of appropriate therapeutic options is severely constrained by this cross-resistance, which in turn renders an increasing number of antibiotics ineffective at treating infections [8,10].

The mechanisms underlying cross-resistance in bacteria are diverse and complex, including efflux pumps, horizontal gene transfer, genetic mutations, and altered target sites [8]. Efflux pumps play an active role in exporting drugs, thereby preventing the accumulation of drugs within the cell, and may cause resistance to multiple antibiotics. Moreover, the induced resistance under the selection pressure of cefotaxime and azithromycin has been reported to cause cross-resistance through AcrB, a component of the AcrAB-TolC tripartite efflux pump system [11]. Similarly, cross-resistance can occur when OmpC is downregulated, as antibiotics from diverse classes enter bacteria through this outer membrane porin [12].

Antibiotic collateral sensitivity is a phenomenon in which resistance to one antibiotic leads to increased susceptibility or hypersensitivity to a different, often structurally unrelated, antimicrobial agent [13]. Unlike antibiotic cross-resistance, collateral sensitivity provides a counterintuitive advantage by capitalizing on bacterial vulnerabilities caused by resistance acquisition [14]. This phenomenon has important implications for antimicrobial research and may open up new avenues for novel therapeutic strategies. Antibiotic collateral sensitivity mechanisms are also diverse and complex [15]. Genetic mutation or modification of bacterial cellular components that confer antibiotic resistance can make the pathogen more susceptible to another. These mutations may disrupt cellular functions, metabolic pathways, or efflux pump systems, resulting in a cascade response of downstream effects that make the bacterium more susceptible to a different antibiotic [14]. Furthermore, changes in bacterial physiology, such as cell membrane permeability or drug target expression, can influence antibiotic collateral sensitivity [15].

Utilizing the potential role of collateral sensitivity in therapies such as combination therapy and alternating therapy can potentially be a successful way to suppress the evolution of resistance or alter the resistance profile [16]. The potential application mechanisms of collateral sensitivity have been studied on several clinically relevant pathogens, including *Escherichia coli* [17,18,19], *Pseudomonas aeruginosa* [20,21], *Enterococcus faecalis* [22], *streptococcus pneumoniae* [23], *Staphylococcus aureus* [24], and *Klebsiella pneumoniae* [15]. For example, the induction of aminoglycoside resistance has been reported to cause increased sensitivity to other classes of antibiotics such as β-lactams, fluoroquinolones, chloramphenicol, tetracyclines, and doxycycline [15,19]. However, such a study on *S*. Typhimurium is very scarce. Therefore, the study was aimed to evaluate the collateral sensitivity and cross-resistance of ciprofloxacin-, gentamicin-, kanamycin-, and tetracycline-induced resistant *Salmonella* Typhimurium ATCC 19585 to cefotaxime, chloramphenicol, ciprofloxacin, gentamicin, kanamycin, polymyxin B, streptomycin, tetracycline, and tobramycin (Table 1).

## 2. Results

### 2.1. Cross-Resistance and Collateral Sensitivity of Antibiotic-Induced Resistant S. Typhimurium

In order to identify cross-resistance and collateral sensitivity, the antibiotic-induced resistant mutants of *S*. Typhimurium ATCC 19585 (ST^WT^) were induced by exposure to ciprofloxacin (CIP), gentamicin (GEN), kanamycin (KAN), and tetracycline (TET), assigned as ST^CIP^, ST^GEN^, ST^KAN^, and ST^TET^, respectively. The antibiotic susceptibility of the induced resistant mutants was determined using cefotaxime, chloramphenicol, ciprofloxacin, gentamicin, kanamycin, polymyxin B, streptomycin, tetracycline, and tobramycin (Table 2). The antibiotic-induced resistant mutants were highly resistant to ciprofloxacin (1024-fold), gentamicin (8-fold), kanamycin (8-fold), and tetracycline (8-fold), respectively.

**Figure 1:**
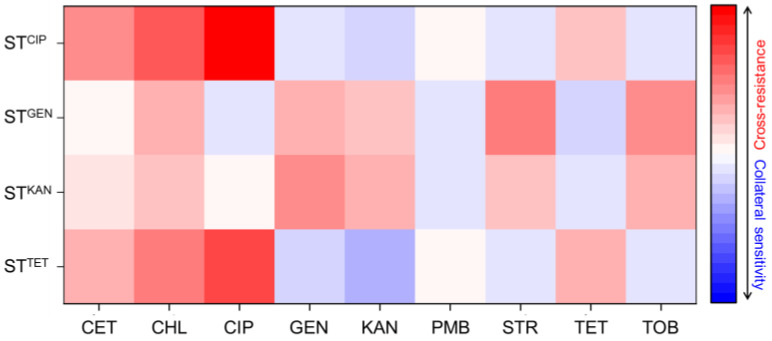
Heat map of fold changes in MIC_R_ of ciprofloxacin-resistant *Salmonella* Typhimurium (ST^CIP^), gentamicin-resistant *S*. Typhimurium (ST^GEN^), kanamycin-resistant *S*. Typhimurium (ST^KAN^), and tetracycline-induced resistant *S*. Typhimurium (ST^TET^) compared to MIC_WT_ of *S*. Typhimurium ATCC 19585 (ST^WT^). Heat map intensities indicate the fold change in MIC compared to that of the untreated ST^WT^; [log_2_ MIC_R_/MIC_W_]. CET, cefotaxime; CHL, chloramphenicol; GEN, gentamicin; KAN, kanamycin; PMB, polymyxin B; STR, streptomycin, TET; tetracycline; TOB, tobramycin.

A heatmap was created to represent the fold change in MICs of ST^CIP^, ST^GEN^, ST^KAN^, and ST^TET^, highlighting both cross-resistance and collateral sensitivity in relation to the wild type across diverse classes of antibiotics (Figure 1). ST^CIP^ was cross-resistant to cefotaxime, chloramphenicol, and tetracycline, ST^GEN^ was cross-resistant to chloramphenicol, kanamycin, streptomycin, and tobramycin, ST^KAN^ was cross-resistant to cefotaxime, chloramphenicol, gentamicin, streptomycin, and tobramycin, and ST^TET^ was cross-resistant to cefotaxime, chloramphenicol, and ciprofloxacin. Surprisingly, ST^CIP^ showed increased susceptibility, and collateral sensitivity, to gentamicin, kanamycin, streptomycin, and tobramycin, ST^GEN^ showed collateral sensitivity to ciprofloxacin, polymyxin B, and tetracycline, ST^KAN^ showed collateral sensitivity to polymyxin B and tetracycline, and ST^TET^ showed collateral sensitivity to gentamicin, kanamycin, streptomycin, and tobramycin. The MIC values of polymyxin B, cefotaxime, ciprofloxacin, and polymyxin B remained unchanged for ST^CIP^, ST^GEN^, ST^KAN^, and ST^TET^.

### 2.2. Role of Antibiotic Resistance Mechanisms in Evolving cross-Resistance and Collateral Sensitivity

The MIC values of ciprofloxacin, gentamicin, kanamycin, and tetracycline, respectively, against ST^CIP^, ST^GEN^, ST^KAN^, and ST^TET^ in the absence and presence of carbonyl cyanide-*m*-chlorophenylhydrazone (CCCP) and phenylalanine-arginine-β-naphthylamide (PAβN) were determined to evaluate the effect of efflux pumps (Table 3). The MIC of ciprofloxacin against ST^CIP^, gentamicin against ST^GEN^, and kanamycin against ST^KAN^ remained unchanged regardless of the presence or absence of CCCP, while the tetracycline resistance of ST^TET^ was increased in the presence of CCCP. Unlike CCCP treatment, the susceptibility of ST^CIP^ to ciprofloxacin was increased in the presence of PAβN. No changes in antimicrobial activities of gentamicin, kanamycin, and tetracycline were observed against ST^GEN^, ST^KAN^, and ST^TET^.

**Figure 2:**
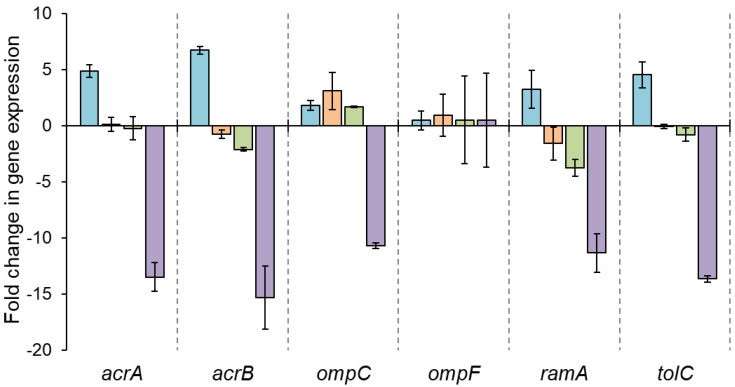
Relative expression of selected genes in ciprofloxacin-resistant *Salmonella* Typhimurium (ST^CIP^, 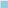), gentamicin-resistant *S*. Typhimurium (ST^GEN^, 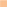), kanamycin-resistant *S*. Typhimurium (ST^KAN^, 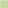), and tetracycline-induced resistant *S*. Typhimurium (ST^TET^, 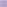).

The relative expression levels of efflux pump- and porin-associated genes were observed in the antibiotic-induced resistant mutants (Figure 2). The relative expression levels of *acrA*, *acrB*, *ramA*, and *tolC* were increased in ST^CIP^. The genes *acrA*, *acrB*, *ompC*, *ramA*, and *tolC*, were suppressed more than 10-fold in ST^TET^.

## 3. Discussion

Bacterial adaptation to a single antibiotic under antibiotic selection pressure may result in enhanced sensitivity to other classes of antibiotics, steered by an evolutionary trade-off between underlying antibiotic resistance mechanisms, termed collateral sensitivity [25]. Collateral sensitivity was first described in the early 1950s by Szybalski and Bryson [26]. The experimentally evolved resistant *E. coli* isolates were less, equally, or more sensitive to antibiotics that were not used for the resistance induction [27]. Although cross-resistance is much more prevalent than collateral sensitivity in antibiotic-resistant bacteria, the phenomenon regarding the collateral sensitivity to antibiotics can provide a possibility of using antibiotics that increase the susceptibility to other antibiotics [14,15,27]. Therefore, collateral sensitivity and its underlying mechanisms have recently been studied in vitro and in vivo levels [15,19,20]. These studies include both Gram-positive bacteria, such as *Staphylococcus aureus* and *Enterococcus faecium*, and Gram-negative bacteria, such as *E. coli* and *Klebsiella pneumoniae* [15,19,28,29]. However, there have been relatively few studies to evaluate the collateral sensitivity in antibiotic-resistant *S*. Typhimurium [25,30].

**Figure 3:**
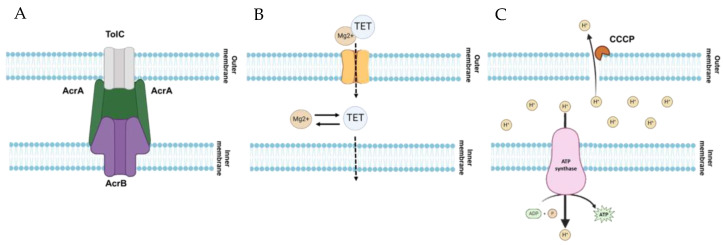
Schematic diagrams depicting AcrAB-TolC efflux pump system (**A**), outer membrane porin (**B**), and proton gradient-induced ATP (**C**).

The collateral sensitivity of bacteria is due to an antibiotic resistance mechanism that can provide a cooperative phenomenon to other classes of antibiotics [31]. For example, the efflux pump-mediated resistance requires increasing proton concentration as the component of proton motive force (PMF) in the bacterial periplasm [32]. On the other side, the penetration of antibiotics such as aminoglycosides into the bacteria is highly dependent on the transmembrane potential, another component of PMF [33]. Thus, the efflux pump-related resistance of bacteria to fluoroquinolone requires a reaching strong PMF, which can induce susceptibility to aminoglycosides. This phenomenon, collateral sensitivity, can possibly be used for re-sensitizing bacteria to antibiotics by reversing multidrug resistance. Ciprofloxacin is an appropriate substrate of AcrAB-TolC efflux pump. AcrAB-TolC, a tripartite resistance-nodulation-division (RND) efflux pump, confers resistance to a broad range of antibiotics [34]. The periplasmic lipoprotein, AcrA, is classified as a fusion protein that bridges the outer and inner membranes [35]. The AcrB is a membrane protein located in the cytoplasmic membrane, and the TolC is an outer membrane protein [36,37]. Together they form AcrAB-TolC tripartite efflux pump system (Figure 3A). This tripartite efflux pump actively extrudes toxic substances, including antibiotics, dyes, disinfectants, and detergents [38]. The efflux pump activity can be reduced by the addition of various natural and synthetic substances called efflux pump inhibitors (EPIs) [39]. CCCP and PAβN are most commonly used as EPIs for experimental purposes.

ST^CIP^ was highly resistant to ciprofloxacin, showing an MIC value of 16 µg/mL (Table 2). The MICs of ciprofloxacin against ST^CIP^ were the same in the absence and presence of CCCP (Table 3). In a recent report, it is found that CCCP might not always be an effective EPI [15]. This is in good agreement with our finding that CCCP did not show reduced efflux pump-mediated resistance (Table 3). Unlike CCCP, the MIC of ciprofloxacin against ST^CIP^ was decreased in the presence of PAβN (Table 3). PAβN is a synthetic efflux pump inhibitor that counteracts the activity of RND family efflux pumps [40]. The modified dipeptide-amide inhibits antibiotic efflux through the AcrAB-TolC system [37]. A computational simulation showed that PAβN binds to AcrB and inhibits efflux pumps at several residues [41,42]. PAβN binds with the proximal substrate-binding site of AcrB to interrupt the antibiotic-AcrB complex formation [43]. The increased susceptibility of ST^CIP^ to ciprofloxacin in the presence of PAβN (Table 3) implies that ST^CIP^ has an RND efflux pump (AcrAB-TolC). Furthermore, the expression levels of *acrA*, *acrB*, and *tolC* were overexpressed in ST^CIP^ (Figure 2). The AcrAB-TolC efflux pump could actively extrude ciprofloxacin out of the bacterial cells, resulting in antibiotic resistance development [37]. The increase in *ramA* expression contributes to the enhanced efflux pump activity by regulating the expression of *acrA*, *acrB*, and *tolC* in *Salmonella* spp. [34]. However, the relative expression levels of *acrA*, *acrB*, *ramA*, and *tolC* were significantly decreased in ST^TET^ (Figure 2).

Tetracycline resistance was less mediated by the AcrAB-TolC efflux pump system than other antibiotic resistance in *S*. Typhimurium [44]. Conversely, the upregulation of AcrAB-TolC was associated with the development of tetracycline resistance in *Klebsiella pneumoniae* [15]. This implies the induction of cross-resistance to ciprofloxacin and tetracycline in antibiotic-resistant *S*. Typhimurium. AcrAB-TolC efflux pump requires PMF as an energy source to extrude antibiotics and other toxic components [39]. The increase in PMF contributed to the increase in bacterial susceptibility to kanamycin, an aminoglycoside antibiotic [45]. On the contrary, the reduced expression of cytochrome oxidases, which plays a vital role in the creation of PMF, induced low membrane potential, and high aminoglycoside resistance [15]. Cytochrome oxidases transport protons from cytoplasm to periplasmic space in the electron transport chain that oxidizes terminal electron acceptors such as oxygen to create PMF [46]. This may explain the increased susceptibility of ST^CIP^ to gentamicin and kanamycin in this study. In Gram-negative bacteria, tetracycline acts as an Mg^2+^ chelator by diffusing through OmpC and/or OmpF [47] (Figure 3B). The major outer membrane porin proteins, OmpC and OmpF, contribute to the accumulation of tetracycline inside the bacterial cells, leading to increased antibiotic susceptibility [48]. In this study, the decreased expression of *ompC* in ST^TET^ might be involved in the increase in tetracycline resistance (Table 2). The ciprofloxacin resistance was also associated with the low expression level of *ompC* in *S*. Typhimurium [49]. Furthermore, tetracycline-resistant Gram-negative bacteria were also sensitive to aminoglycosides, which is in good agreement with the finding of this study [15,19].

PMF consisting of transmembrane electrical potential (Δψ) and transmembrane proton gradient (ΔpH) plays a major role in aminoglycoside internalization into bacterial cells [33,50]. Bacteria maintain the electrochemical potential balanced by Δψ and ΔpH in the cytoplasmic membrane [50,51]. Thus, the perturbation of PMF may result in compensatory phenomena in bacteria [52]. The membrane potential-uncoupling antibiotics may collapse PMF due to the dissipation of Δψ or ΔpH [52]. Unlike ATP-binding cassette (ABC) transporter, the multidrug efflux pump families such as RND, small multidrug resistance (SMR), multidrug and toxic compound extrusion (MATE), and major facilitator superfamily (MFS) require PMF that is generated by cellular metabolism [53,54,55]. The perturbation of PMF can cause a decrease in the activity of PMF-dependent efflux pumps and result in an increase in antibiotic susceptibility [19]. A protonophore, CCCP, disrupts the PMF to reduce/abolish the activity of efflux pumps [40] (Figure 3C). The decrease in membrane potential, consequently PMF, is responsible for the increased resistance to aminoglycoside [15]. The cross-resistance of ST^GEN^ and ST^KAN^ to gentamicin and kanamycin and collateral sensitivity to ciprofloxacin and tetracycline may be attributed to PMF dissipation. The decreased PMF in ST^GEN^ and ST^KAN^ might decrease the activity of AcrAB-TolC, leading to increased sensitivity to ciprofloxacin and tetracycline.

It is important to recognize some limitations in our study of induced cross-resistance and collateral sensitivity in *S*. Typhimurium. First of all, whole-genome sequencing was not performed, which could have given a thorough understanding of the genetic basis of the observed changes in sensitivity and resistance. Due to this reason, the precise genetic mutations or modifications in metabolic pathways that may be responsible for these phenomena could not be addressed in this article. In addition, not all known potential resistance mechanisms were examined in the study. Numerous genetic, biochemical, and physiological factors may play a role in the complex process of antibiotic resistance in *S*. Typhimurium and can lead to the emergence of cross-resistance as well as collateral sensitivity. While some well-known mechanisms have been explored, there might be other potential mechanisms that were not investigated in this study. Despite the limitations, this study provides new useful information on antibiotic-induced cross-resistance and collateral sensitivity in *S*. Typhimurium. The results may serve as the foundation for follow-up research, which may include whole-genome sequencing and a thorough examination of antibiotic resistance mechanisms to gain a better understanding of the complexities of antibiotic resistance in *S*. Typhimurium. These studies would aid in the development of effective anti-antibiotic resistance strategies and enhance the treatment options for *Salmonella* infections.

## 4. Materials and Methods

### 4.1. Strain and Culture Conditions

*Salmonella* Typhimurium ATCC 19585 (ST^WT^) was purchased from American Type Culture Collection (ATCC, Manassas, VA, USA). The strain was cultured for 18 h at 37 °C in trypticase soy broth (TSB; BD, Becton, Dickinson and Co., Sparks, MD, USA) supplemented with 0.1% yeast extract (TSBY). The culture was collected by centrifugation at 6000× *g* for 10 min at 4 °C. The harvested cells were then washed twice with phosphate-buffered saline (PBS, pH 7.2) prior to use.

### 4.2. Preparation of Antibiotic Stock Solutions

The antibiotics used in this study (Table 1) were purchased from Sigma Chemical Co. (St. Louis, MO, USA). The stock solutions of cefotaxime (water), chloramphenicol (ethanol), ciprofloxacin (glacial acetic acid), gentamicin (water), kanamycin (water), polymyxin B (water), tetracycline (ethanol), streptomycin (water), and tobramycin (water) were prepared by dissolving in appropriate solvents at a concentration of 10.24 mg/mL.

### 4.3. Induction of Antibiotic-Resistant Salmonella

ST^WT^ was used to induce antibiotic resistance to ciprofloxacin (ST^CIP^), gentamicin (ST^GEN^), kanamycin (ST^KAN^), and tetracycline (ST^TET^) according to a previous method [56] with slight modification. In brief, ST^WT^ was cultured in TSBY with 1/2×MIC of the above-mentioned antibiotics individually for the first passage. After 24–72 h of incubation at 37 °C, the cultures (200 µL each) were transferred to TSBY with A 2-fold increase in the concentration of the same antibiotic and then serially incubated at the same condition. This procedure was continued until no growth was observed after 72 h of incubation.

### 4.4. Antibiotic Susceptibility Assay

The effects of efflux pump inhibitor phenylalanine-arginine-β-naphthylamide (PAβN) and protonophore carbonyl cyanide-*m*-chlorophenylhydrazone (CCCP) on antibiotic susceptibility of ST^WT^, ST^CIP^, ST^GEN^, ST^KAN^, and ST^TET^, were evaluated using a broth microdilution susceptibility assay according CLSI guideline [57]. In brief, approximately 10^5^ CFU/mL of ST^WT^ and the antibiotic-induced resistant mutants were inoculated in the 96-well plates containing serially diluted (1:2) stock solutions from 1024 µg/mL in TSBY. The prepared plates were incubated at 37 °C for 18 h to determine MICs of antibiotics in the absence and presence of PAβN and CCCP.

### 4.5. RT-qPCR Assay

Total RNAs in ST^WT^ and the antibiotic-induced resistant mutants were extracted by using the RNeasy Protect Bacteria Mini kit (Qiagen, Hilden, Germany). The RNA extracts were mixed with 1 mL of RNAprotect Bacteria reagent (Qiagen) to prevent RNA degradation, centrifuged at 5000× *g* for 10 min, and then lysed by TE buffer containing lysozyme (1 mg/mL). The lysates were mixed with ethanol to purify RNA using an RNeasy mini column (Qiagen). The RNAs were quantified using a NanoDrop spectrophotometer. Complementary DNA (cDNA) was synthesized by transcribing RNA templates through QuantiTect Reverse Transcription kit (Qiagen). In brief, the purified RNA was reacted with a Wipe buffer to eliminate genomic DNA (gDNA). The reactants were mixed with a Reverse Transcriptase (RT) Master mix containing RT Buffer, RT Primer Mix, and Reverse Transcriptase. The mixtures were reacted for 15 min at 42 °C and inactivated at 95 °C for 3 min. The PCR mixture (20 μL) was prepared by mixing 2 μL of cDNA with 1.2 μL of reverse and forward primers, 10 μL of SYBR Green, and 5.6 μL of Nuclease-free water and reacted through a QuantStudio™ 3 Real-Time PCR System (Applied Biosystems™, MA, USA). The thermal cycling conditions for the qPCR assay were set at 45 cycles. The PCR mixture was denatured at 95 °C for 5 s, annealed at 55 °C for 20 s, and extended at 72 °C for 15 s. The primers, including reference gene, multidrug efflux pump, outer membrane porin, and transcriptional activator-associated genes, are listed in Table 4. The gene expression was relatively estimated by comparing Ct values according to the comparative method [58]. The expression levels of target genes (*acrA*, *acrB*, *ompC*, *ompF*, *ramA*, and *tolC*) in the antibiotic-induced resistant mutants relative to ST^WT^ cells were estimated and calculated using the formula; ΔΔCt = ΔCt_treatment_ − ΔCt_calibrator_. The ΔCt_treatment_ is the Ct values for the antibiotic-induced resistant mutants were normalized to 16S rRNA (Ct_treatment_ − Ct [16S rRNA]_treatment_), and the ΔCt_calibrator_ is the Ct values for the ST^WT^ cells normalized to 16S rRNA (Ct_calibrator_ − Ct [16S rRNA]_calibrator_). The comparative ΔΔCt method was validated by the amplification efficiencies of the respective target genes (*acrA*, *acrB*, *ompC*, *ompF*, *ramA*, and *tolC*) and the reference gene (16S rRNA).

## 5. Conclusions

Collateral sensitivity has important clinical implications and may transform the approach to antibiotic therapy. By strategically combining antibiotics with collateral sensitivity, it is possible to induce the enhanced susceptibility of bacteria to antibiotics, enhancing bacterial eradication and lowering the risk of resistance development. This concept has great potential not only in human medicine but also in veterinary and agricultural settings, where multidrug-resistant pathogens pose a significant threat to animal health and food production. This study describes cross-resistance and collateral sensitivity in ST^CIP^, ST^GEN^, ST^KAN^, and ST^TET^ in association with the induction of antibiotic resistance. The most significant finding of this study was the collateral sensitivity of ST^CIP^ and ST^TET^ to gentamicin and kanamycin. ST^KAN^ showed no collateral sensitivity to ciprofloxacin. The efflux pumps, and outer membrane porin proteins were linked to the collateral sensitivity and cross-resistance. These results can be useful for designing effective antibiotic treatments, such as alternating and combination antibiotic treatments for infections caused by antibiotic-resistant bacteria. Further study is needed to investigate whole genome sequencing of the antibiotic-induced resistant mutants to elucidate the exact genetic alteration responsible for increased antibiotic susceptibility. In addition, a gene knockout and complementation study is underway in our lab to validate the mechanisms associated with collateral sensitivity.

## Figures and Tables

**Table 1:** Antimicrobial properties of antibiotics used in this study.

Class	Antibiotic	Target Site	Antimicrobial Activity
Cephems	Cefotaxime	Cell wall	Bactericidal
Phenicols	Chloramphenicol	50S ribosomal subunit	Bacteriostatic
Fluoroquinolones	Ciprofloxacin	DNA gyrase	Bactericidal
Aminoglycosides	Gentamycin	30S ribosomal subunit	Bactericidal
Aminoglycosides	Kanamycin	30S ribosomal subunit	Bactericidal
Aminoglycosides	Streptomycin	30S ribosomal subunit	Bactericidal
Aminoglycosides	Tobramycin	30S ribosomal subunit	Bactericidal
Glycopeptides	Polymyxin B	Cell membrane	Bactericidal
Tetracyclines	Tetracycline	30S ribosomal subunit	Bacteriostatic

**Table 2:** Minimum inhibitory concentrations (MICs; µg/mL) of selected antibiotics against *Salmonella* Typhimurium ATCC 19585 (ST^WT^), ciprofloxacin-resistant *S*. Typhimurium (ST^CIP^), gentamicin-resistant *S*. Typhimurium (ST^GEN^), kanamycin-resistant *S*. Typhimurium (ST^KAN^), and tetracycline-induced resistant *S*. Typhimurium (ST^TET^).

Antibiotic	ST^WT^	ST^CIP^	ST^GEN^	ST^KAN^	ST^TET^
Cefotaxime	0.0625	1	0.0625	0.125	0.5
Chloramphenicol	0.5	32	4	2	16
Ciprofloxacin	0.0156	16	0.0078	0.0156	2
Gentamicin	8	4	64	128	2
Kanamycin	32	8	128	256	4
Polymyxin B	4	4	2	2	4
Streptomycin	32	16	1024	128	16
Tetracycline	2	8	0.5	1	16
Tobramycin	16	8	256	128	8

**Table 3:** Minimum inhibitory concentrations (MICs; µg/mL) of ciprofloxacin, gentamicin, kanamycin, and tetracycline of ciprofloxacin-resistant *Salmonella* Typhimurium (ST^CIP^), gentamicin-resistant *S*. Typhimurium (ST^GEN^), kanamycin-resistant *S*. Typhimurium (ST^KAN^), and tetracycline-induced resistant *S*. Typhimurium (ST^TET^) in the absence and presence of CCCP and PAβN.

Strain	Antibiotic	Efflux Pump Inhibitor		
		No	CCCP	PAβN
ST^CIP^	Ciprofloxacin	16	16	2
ST^GEN^	Gentamicin	64	64	64
ST^KAN^	Kanamycin	256	256	256
ST^TET^	Tetracycline	32	64	32

**Table 4:** Primers used for real-time RT-PCR analysis.

Gene	Molecular Function	Primes Sequence	References
16s rRNA	Reference gene	F: AGGCCTTCGGGTTGTAAAGT R: GTTAGCCGGTGCTTCTTCTG	[59]
*acrA*	Multidrug efflux pump	F: AAAACGGCAAAGCGAAGGT R: GTACCGGACTGCGGGAATT	[59]
*acrB*	Multidrug efflux pump	F: TGAAAAAAATGGAACCGTTCTTC R: CGAACGGCGTGGTGTCA	[59]
*ompC*	Outer membrane porins	F: TCGCAGCCTGCTGAACCAGAAC R: ACGGGTTGCGTTATAGGTCTGAG	[60]
*ompF*	Outer membrane porins	F: CGGAATTTATTGACGGCAGT R: GAGATAAAAAAACAGGACCG	[60]
*ramA*	Transcriptional activator	F: CCAGAAGGTGTATGATATTTGTCTCAAG R: GGTTGAACGTGCGGGTAAA	[60]
*tolC*	Multidrug efflux pump	F: GCCCGTGCGCAATATGAT R: CCGCGTTATCCAGGTTGTTG	[59]

## Data Availability

Data available on request.
